# Extraction and chemical characterization of humic acid produced from lignite coals of arid region of Gujarat, Western India

**DOI:** 10.1038/s41598-024-81861-6

**Published:** 2024-12-28

**Authors:** Juhi Rani, Sushmita Kumari, Biswajit Paul

**Affiliations:** Department of Environmental Science and Engineering, Indian Institute of Technology (Indian School of Mines), Dhanbad, Jharkhand 826004 India

**Keywords:** Lignite, Humic acid, Extraction, Elemental analysis, Characterization, Environmental sciences, Energy science and technology

## Abstract

In the current study, extraction of humic acid (HAs) from lignite fines of kutch basin of Gujarat (western India) were reported. The extraction was done by International Humic Substances Society (IHSS) method. Several analytical and spectroscopic techniques were used to characterize of extracted HAs. The gravimetric analysis showed (28.5% and 26.4%) of humic acid extraction from panandhro mines (PM) and mata-No-Madh mines (MNMM) respectively. UV–Vis spectroscopy revealed a high degree of humification, higher stability and aromatic nature. FTIR peaks demonstrated the presence of carboxylic, alcoholic, and phenolic functional groups. SEM/EDX analysis revealed that surface were smooth, non-porous and like loose sponge that showed the presence of major elements like C, O, F, Al, Na, S, Si, Ca, Ti and Fe. The combination of results gives a better and improved understanding of the nature of lignite coals of western India. It may be helpful in choosing suitable coals for the extraction of humic acid and using it for enhancing plant growth condition, soil enrichment and creating green energy solution. This study not only helps in the research related to extraction of humic acid from lignite but also creates a new avenue for the efficient and clean use of lignite.

## Introduction

Coal is known as the combustible sedimentary rock. It is formed by the accumulation and decomposition of plant and animal residues by the process known as coalification^1^. It is of four types namely anthracite, bituminous, lignite and peat on the basis of degree of coalification, heating value, fixed carbon content and ash content^2^. Anthracite and bituminous are high-rank coals (HRCs) and lignite (brown coal) and peat coals are low rank coals (LRCs). Lignite is an intermediate product between bituminous and peat coal and it is used as a fuel for power plants. In the world, lignite makes up almost 45% of all coal reserves^3^. It has high content of moisture (> 30%), less heating value (< 19.3 kJ kg^−1^), more ash (up to 25%), volatile content (more than 24%) and higher level of CO_2_ emission factor (> 0.365 kg CO_2_-eq/MWh). However, the usage of lignite is limited to the production of power because of its qualities, which include a high ash concentration and high level of impurities. Due to these factors, power plants that uses lignite as a fuel emits more CO_2_ that lessen the power generation efficiency and increases the consumption of fuel^4,5^.

India ranks third in the world for lignite and coal production. The two stratigraphic horizons that the Indian coal deposits are linked to are the tertiary coal and lignites of the Palaeogene period and the gondwana coal of the Permian age^6^. The significant and biggest lignite deposits in India are present in the state of Tamilnadu, Gujarat and Rajasthan. In the state of Gujarat, Kutch, Cambay and Saurashtra basins have extremely thick lignite deposits. The Gujarat Mineral Development Corporation (GMDC) mines the lignite in the Kutch basin in the Panandhro and Mata-no-madh areas. Lignite mines are mostly operated by opencast method of mining. During the mining process, numerous machinery are utilised in the excavation, overburden (OB) removal and transportation activities. These mechanized processes and the overuses of the mechanical tools leads to the generation of coal fines. In addition to that, it is also generated due to some natural friability and during the process of handling and size reduction methods^7,8^. There is a difficulty in treatment of these coal fines. So, it is either added to steam coal or get disposed of and abandoned on stockpiles and slurry ponds closer to the mining sites irrespective of the fact that it represents a highly promising energy source^9^. This remaining source of energy has been gaining attention recently due to increasing energy prices, increasing coal extraction costs, increased environmental problems (combustion, emission of sulphur dioxide and carbon dioxide, acid mine drainage), decrease in the amount of land available for clearing coal mine waste and challenges in obtaining thermal coal of a higher grade^10^. Therefore, in recent years the proper use of coal fines has become a difficult task and it is overcome by more efficient, environmentally friendly and sustainable routes^11^. In order to produce products with a greater value added, humic acids (HAs) extraction that is a major fraction of humic substances that is soluble in alkaline solutions and insoluble in acidic mediums, has emerged as a potential hotspot for academic research teams and various industries.

Humic acids (HAs) are polyhydroxy carboxylates which have both aromatic and aliphatic subunits^12^. It is light brown to the black in colour. It has complex chemical structure and hydrophilic, aromatic and acidic characteristics. It has various functional groups like carboxylic, phenolic, ketones and hydroxyl groups. It is a complex chemical substance, so it is essential to break it into simpler compound so that the analysis got easier using traditional methods. Some of divided compound can act as a useful product. Strong alkali treatment that separates the three components (alkali-insoluble, alkali-soluble and acid-insoluble, alkali- and acid-soluble) is the most widely used method for fractionating lignite. Humic acids are alkali-soluble, acid-insoluble fraction that makes up between 10 and 80% of the organic content in lignite^12^. The percentage of organic matter depends upon the maturity of organic matter. Sometimes the structure helps to know about the characteristics and nature of original lignite. The quantity of extractable HAs based on the method of alkaline extraction depends on the type and concentration of the extractant, raw material’s particle size, the temperature and duration of the reaction, the ratio of coal to water, and the extraction process^13^. HAs are most commonly employed in agricultural applications as an organic fertiliser and feed supplement^14^. In agricultural field, it is known as “Black Gold” of agriculture. It plays an essential role in ecological processes as it is a source and sink of carbon for the atmosphere and the biosphere. It also acts as a source and sink of fertilisers for plants and a source of dietary supplements for agriculture uses^15^. As the mineralization of humic substances releases NH_4_^+^, CO_2_, NO_3_^−^, SO_4_^2−^, PO_4_^3−^ that is very important nutrient source for growth of plant. It increases the rate of seed germination, stimulation in root-shoot growth, increase in enzymatic activity and resilience against abiotic stressors such as high salinity and pH^16^. It also plays crucial role in global carbon and nitrogen cycle, complexation of minerals and oxidation–reduction reaction^2^. It is also used in the field of biomedical/nutraceuticals uses^17^, additive for curing rubber, colouring, lithium-ion batteries industries^18^ and ruminant feed supplements^19^. It has a huge possibility in the field of nanotechnology^20^.

The International Humic Substance Society’s (IHSS) extraction method is often the most widely used technique for extracting HAs. Additionally different methods are also used like conventional extraction method, process intensification method (includes ultrasound, microwave, hydrodynamic cavitation and so on fungal/microbial based production method), oxidation pre-treatment method (includes chemical HNO_3_/H_2_O_2_ based oxidation and gaseous methods (includes air/oxygen/ozone based oxidation)^21–24^. Different extraction methods are used for the extraction of HAs from different raw material. The % of extractable HAs from various raw materials such as leonardite, lignite, brown coal and peat are 40–90, 30–80, 10–20 and 10–25% respectively^24^. Table 1 shows the extraction of HAs and its degree of purity from various raw materials by using different extraction and purification technology.

**Table 1:** The degree of purity of HA produced by utilising various raw materials by different extraction and purifying techniques.

S. no	Reference	Raw material	Region	Purity of HA	Extraction and purification technology
1.	Chen^25^	Lignite	China	95%	Alkaline extraction, membrane dialysis and freeze drying
2.	Zara et al.^23^	Lignite	Pakistan	13–29%	Alkaline extraction using International protocol
3.	Prosyolkov et al.^26^	Brown coal	Russian	90,70 and 40%	Ultrasound, alkaline protocol using reactor and multiple filtration
4.	Novak et al.^27^	Brown coal	Czeck Republic	Not determined	Membrane ultrafiltration and alkaline extraction
5.	Shakiba^28^	Leonardite	Iran	24%	Filter press system and alkaline extraction
6.	Ghani et al.^2^	Low grade Thar	Pakistan	Not determined	Alkaline extraction using International protocol
7.	Nasir et al.^12^	Lignite	Pakistan	18%	Alkaline extraction using IHSS

Several studies have been done regarding the extraction of HAs in different parts of the world. Zara et al.^23^ conducted an experiment on the extraction of HAs from low rank Pakistani lignite coal from Thar, chakwal and Quetta region. The gravimetric method showed that there were 24.6, 13.6, and 18.0% of humic acid extraction from Thar, Chakwal, and Quetta coals respectively. FTIR analyses of extracted humic acid samples have shown the presence of carboxylic, phenols, alcoholic, and amines functional groups. The result concluded that the study could be useful for selection of suitable coals for humic acid extraction. Wali et al.^29^ investigated the efficient extraction of humic acid from Tunisian lignite and the characterization showed the predominance of OH, COOH, CH, C=C and COO groups, which are typical functional groups in humic substances. The effect of produced new humic acid (HA) was seen on wheat germination and root elongation. The findings demonstrated that a significant germination percentage was achieved by wheat seeds germinating in HA fertiliser that had been diluted 20 times. Indeed, the addition of diluted HA caused the wheat root’s elongation rate to increase by 120% in comparison to the controls. Souza and Braganca^30^ conducted an experiment on the extraction of humic acid from sub-bituminous coal to be applied as ceramic dispersant. The HA was characterized via elemental analysis, TGA/DTA, FTIR, zeta potential/turbidity and SEM. Phenolic and carboxylic group were identified and the structure and surface properties were related to the polyelectrolytic nature of humic acid. The resultant humic acid acts as a good dispersant for ceramic industry according to the result of the rheological investigation in an alumina suspension. There was a significant decrease in suspension viscosity while using humic acid and the values are compatible to those necessary for ceramic processing. In the year 2006 in India, a process for the production of ‘’Humi Gold’’ (a salt of humic acid) has been patented by nevyeli lignite corporation, India^31^. The patent describes the process of production of potassium salt of humic acid by extraction using aqueous potassium hydroxide of lignite. Thus, the obtained potassium humate is known as ‘’Humi Gold’’ that is resulted as a black flakes. It is used for promoting growth on groundnut, maize and ladies finger^31–33^. The lignite from nevyeli mines has been considered as a potential source for the new chemical with possible technological significance. Mukherjee et al. (2010) conducted an experiment on the solubilisation of nevyeli lignite by oxidative degradation. The research was guided in such a way that in the future there will be scarcity of petroleum derived hydrocarbons for use as a starting material for the synthesis of organic chemicals. This shortage has renewed interest in the use of coal/lignite as a raw material for chemical production. The remarkable rise in lignite’s solubility in organic solvent following treatment with diluted nitric acid was thought to be an immediate pathway for its direct utilization. The study was aimed at selection of the required reaction parameters for the conversion of lignite to the source of carbochemicals. The results were analysed by various chemical and spectroscopic techniques and concluded that substances possess both aromatic and aliphatic characteristics. The dominant functional groups which contribute to the reactivity of the substance are phenolic and carboxylic acid. Therefore, all these findings suggested that there is high potential of extraction of humic acid from coal/lignite. There were various published literature and patents worldwide on the extraction of humic acid by different techniques and its uses in a variety of way. But as per author’s knowledge there is no any such kind of research or theoretical work happening in lignite mining area of Kutch, Gujarat (India). There is a big research gap in this area related to such study. Therefore, the present study is taken with the objective of extraction and characterization of humic acid from two lignite fields of Kutch basin of India by using IHSS method. The characterization are done by various analytical methods including CHNOS elemental analysis, and their O/C, H/C and C/N atomic ratios, acidic functional groups titration analysis, ultraviolet–visible (UV–Vis), fourier-transform infrared radiation (FT-IR), scanning electron microscopy (SEM) along with energy dispersive X-ray (EDX)^34^. These methods have been widely used to investigate the chemical, spectral and microstructural properties of HAs. Furthermore, the study also compares the yield, the structural and chemical properties of the extracted HAs from the two fields. This type of study is helpful in the way that the produced humic acid is used as soil conditioning agents that boost agricultural productivity and add values to the low rank coal.

## Materials and methods

### Materials

The lignite samples were collected from the panandhro mines (PM) and mata-no-madh mines (MNMM) of Kutch district of Gujarat, India by bulk sampling. A representative sample was taken from the bulk sample for detailed investigation. At 40 °C the sample were dried and grounded in a quartz mortar. After that it was stored in a sealed plastic flask at 4 °C to minimise undesirable oxidation. Analytical grade chemicals such as hydrochloric acid (HCl), Sodium hydroxide (NaOH), hydrogen fluoride (HF) were commercially purchased. Ultra-pure water (Milli-Q system Millipore) was used for the dilution and dialysis purpose of extracted samples. In order to compare laboratory extracted humic acid with standard humic acid that is represented as STD-HAS, the humic acid of sigma Aldrich grade (Cas No-1415-93-6, Ec No-215-809-6) was purchased. The extracted humic acid from the lignite sample of Panandhro and mata-no-madh mines are represented as P-HAS and M-HAS.

### Humic acid extraction

#### Alkaline extraction followed by acidic pre-treatment

**Fig. 1:**
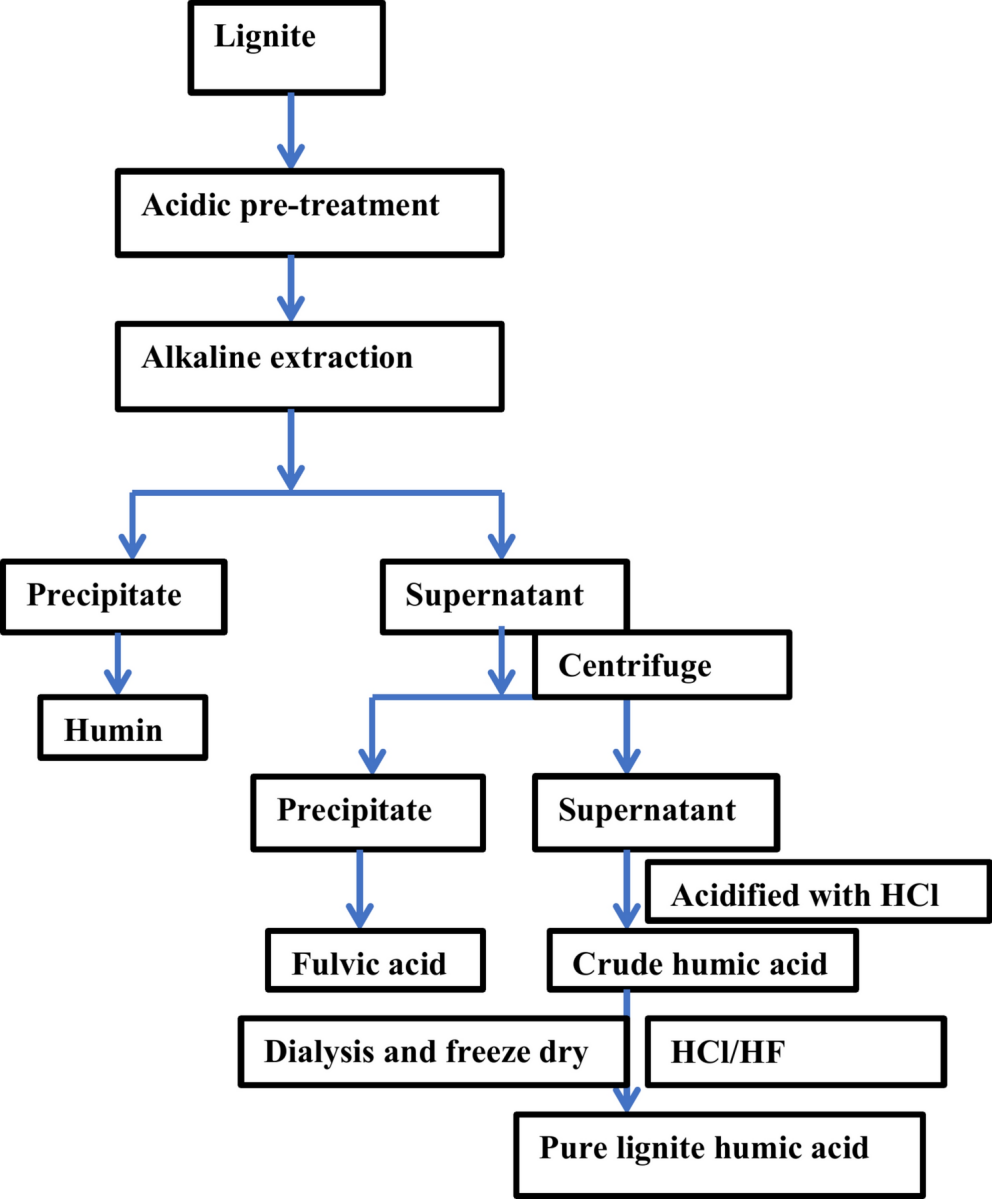
Schematic flow sheet for extraction of lignite humic acid.

The method of International Humic substances society (IHSS) was used for the isolation and purification of humic acid that was followed by acidic pre-treatment. The acidic pre-treatment make the extraction of humified matter easier. 0.1 g of coal fine samples were taken and pre-treated with 100 mL of 0.1 mol L^−1^ HCl and shaken for 2 h and 30 min. The acidic supernatant was separated by the process of centrifugation at 2000 rpm for 10 min after that it was discarded and the process was repeated up to three times. The alkaline extraction was done after the acidic pre-treatment. The extraction was carried out by mechanically shaking the sample in 30 mL of 0.5 mol L^−1^ NaOH for 3 h. Alkaline suspension was allowed to settle overnight and the sample was centrifuged for 30 min at 4000 rpm for the collection of supernatant. Later the separation process takes place that separates the humic and fulvic acid. It was done by addition of 0.1 mol L^−1^ HCl to the supernatant until the pH of the solution will reach 2 and it was left to stand for 24 h. After that, the centrifugation was done to separate the humic acid (precipitate) fraction. Further the purification step takes place and the primary objective of purification is to minimize the weight of ash, while the secondary objective consists of separating the organic molecules of lower molecular weight of humic materials^35^. The procedure involved treating the sample with diluted solution of 5% HCl/HF solution in a 1:1(v/v) ratio to the HA in a mechanical shaker for two hours. It was done to reduce the weight of the ashes. Schnitzer^36^ noted that HCl-HF treatment can reduce the ash content to less than 1%. The purified humic acid was washed with deionized water for three times. It was then frozen overnight and kept in a vacuum oven at 60 °C till drying. After that the dried samples were collected and kept in a desiccator with silica gel for further characterization^37^. The schematic flow sheet for extraction of humic acid are shown in Fig. 1. The yield of humic acid was determined through gravimetric analysis. For gravimetric analysis 0.001 M diethylenetriaminepenta acetic acid (DTPA) solution was prepared. For the preparation of 0.001 M DTPA solution, 0.0393 g of DTPA was used. The 1000 mL reagent solution was made with deionized water by the addition of 0.001 M DTPA solution in 1 L and the addition of 40 g of NaOH and 20 mL of ethanol was done before saving it for gravimetric analysis. For the gravimetric analysis, 100 mL of reagent solution as mentioned above was taken and mixed with 5 mL of extracted humic acid solution in a flask. The mixture was then shaken in a mechanical shaker for 1 h at 200 rpm. After that, the concentrated nitric acid was added till pH 1.0 was obtained. The mixture was then left as such for 2 h to complete the reaction. After completion of reaction, humic acid precipitates was filtered through whatman filter paper no 42 and oven dried at 105 °C^23^. The percentage content of humic acid was determined by using Eq. (1)

1 $$\text{HA }(\text{w}/\text{v},\text{\%})= \frac{weight\, of\, oven\, dried\, precipitates}{volume\, of\, humic \,acid \,sample\, taken}\times 100.$$

#### Proximate analysis

The proximate analysis make up of volatile matter (VM), moisture content (MC), ash content (AC) and fixed carbon (FC). The determination of moisture content was done by drying the sample to a constant mass at 105 °C in an oven overnight. The volatile matter was determined by drying the sample in a muffle furnace at temperature of 950 °C and then the difference was calculated in weight percent loss during combustion as per standard (ASTM, D3175) method. The ash content was determined by combustion of samples in a muffle furnace at the temperature of 750 °C for 4 h (ASTM, D3174). Fixed carbon was calculated by adding the total percentage of content of volatile matter, ash content, and moisture content and subtracted it by total percentage.

### Elemental analysis

The determination of the elemental composition of lignite fines and P-HAS and M-HAS was done by CHNS Unicube model analyser (Elementar Analysen systeme GmbH, Germany). The carbon (C), hydrogen (H), nitrogen (N) and sulphur (S) were directly taken from the analysis and the determination of the amount of oxygen (O) was calculated by formula i.e. % O = 100% − (%C + %H + %N + %S) after taking into account the ash yield of lignite and derived materials. The elemental analysis of all the samples was summarized in Table 2. On the basis of elemental value, the atomic ratio H/C, O/C and C/N were calculated. Mineral matter (MM) content was calculated using the Parr formula:

$${\text{MM}} = { 1}.0{8} \times {\text{ash}} + 0.{55} \times {\text{sulphur}}.$$

**Table 2:** Proximate analysis of the samples (wt%).

S. no	Samples	Proximate analysis (%)			
		MC	VM	AC	FC
1	PM	31.2	37.2	13.3	18.3
2	MNMM	36.4	29.1	24.00	10.5
3	P-HAS	9.3	10.5	2.5	4.3
4	M-HAS	8.2	11.7	1.8	3.8

*MC* moisture content, *VM* volatile matter, *AC* ash content, *FC* fixed carbon.

The calculation of internal oxidation degree (ω) was done on the basis of elemental composition according to Eq. (2) that is given by^38^

2 $$\omega \, = \, \left( {{\text{2O}} + {\text{3N}} - {\text{H}}} \right)/{\text{C}},$$

where O, N, H, C are elemental composition in atomic percentages.

On the basis of Eq. (3) given by Saikia et al.^39^, the aromaticities (f_a_) of the samples were determined.

3 $${\text{f}}_{{\text{a}}} = \, 0.0{35 }\left( {{\text{C}}/{\text{H}} + \, 0.0{57}} \right) \, + \, \left( {0.{175} + 0.0{22}} \right).$$

### Functional group determination

#### Total acid group determination

The total acid group determination was done by the barium ion-exchange method. It was originally given by (Schafer,1970) which was further modified by Allardice et al.^40^ in the late 1990s. In an experiment 250 mg of freeze-dried sample was suspended in 50 mL of 0.2 mol BaCl_2_/Ba (OH)_2_ solution. After that, stirring was done for 30 min under reduced pressure. Then, it was filtered and rinsed with 5 mL BaCl_2_/NaOH solution for three times. The sample was then suspended in 0.2 mol L^−1^ HCl (10 mL) and stirred for 30 min under vacuum condition and then it was filtered.

#### Carboxylic acid determination

The similar procedure was used for the determination of carboxylic acid (–COOH) as used for the total acid group determination only the difference is in the uses of exchange solution i.e. (BaCl_2_/N(CH_2_CH_2_OH)_3_/HCl (60 mL) was used instead of BaCl_2_/Ba (OH)_2_ (50 mL) distilled water was used as a rinsing agent instead of BaCl_2_/NaOH.

#### Phenolic group determination

The phenolic group was determined by calculating the difference between the acidity of the carboxylic groups and the total acidity.

### Spectroscopic techniques

#### Ultraviolet–Visible spectroscopy (UV–Vis)

Ultraviolet–Visible spectroscopy (UV–Vis) of the extracted HAs and the standard HAs was conducted by Agilent Cary 5000 in the range of 175–3300 nm using tungsten-halogen and deuterium light source. 2–4 mg of humic substance was dissolved in 10 mL of 0.05 mol (NaHCO3) L^−1^ solution for the absorbance measurement. After proper dissolution, the pH of the solution should be maintained close to 8 (0.05 mol (NaHCO_3_) L^−1^ aqueous solution has a pH 8.3). The reference tank was filled with a pure 0.05 mol (NaHCO3) L^−1^ solution, and the quartz tank is filled to mid-height with the dissolved solution. The absorbance was recorded at wavelength of 250–800 nm. The E_4_/E_6_ ratio (Abs_465_/Abs_665_) and the value of Log K (log Abs_465_ − log Abs_665_) were calculated using the absorbance measurements at 465 and 665 nm for the characterization of HAs^41,42^.

#### Fourier-transform infrared spectroscopy (FTIR)

The Fourier-transform infrared spectroscopy (FTIR) is the way to learn about the configuration of the functional and structural properties in the HAs at specific vibrations. It acts as a very helpful tool for the preliminary characterization of humic materials of various origins. It is also used to assess how different extractions or chemical purification techniques affect the structure of the prepared humic acid. The spectra were recorded from Invenio S, Bruker Optik; GmBh. KBr pellet is required for the FTIR analysis. The preparation of pellet was done by mixing thoroughly 2 mg of humic acid with 200 mg dried KBr utilizing hydraulic press.

#### Scanning electron microscope (SEM) along with energy dispersive X-ray (EDX)

The structural and chemical characterization of the HAs was studied by the technique of scanning electron microscopic technique (SEM). The SEM model (FE-SEM Supra 55: Carl Zeiss, Germany, Schottky Field Emission Electron Gun: Resolution: 0.8 nm at 15 kV, 1.6 nm at 1 kV, Acceleration voltage: 0.02 V to 30 kV with gun type) was used for the characterization. Along with SEM, Energy Dispersive X-ray analysis (EDX) that has Silicon Drift Detector (SDD) sensor, throughput > 200,000 cps, resolution: @ 127 eV @ MnKα, count rates > 500,000 cps) was used. It is used to know about the surface morphologies and chemical composition of the extracted material.

## Results and discussion

### Humic acid yield

**Fig. 2:**
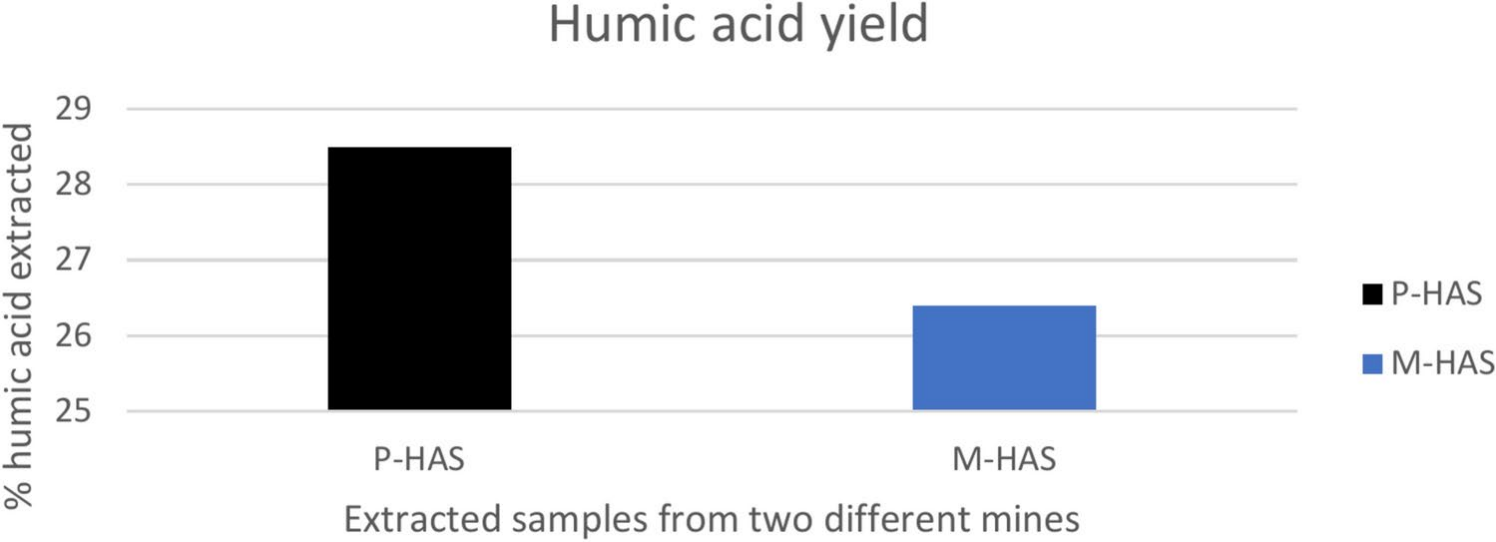
Graph of the (%) yield of extracted HAs (P-HAS and M-HAS) from two different lignite mines.

The humic acid yield is dependent on extraction, refinement procedure and raw material rather than the humic acid content in lignite. In the present study, the extraction of HAs takes place from the two different lignite samples of Kutch basin i.e., Panandhro and Mata-no-Madh mines i.e., named as P-HAS and M-HAS. The yield of both the samples was calculated based on the Eq. (1). The calculated yield of P-HAS and M-HAS were 28.5% and 26.4% respectively that is shown in Fig. 2. The % yield of both the samples was almost similar and low and it may be due to similarity in extraction and refinement procedure and high ash content in lignite. A study was done on the extraction of humic acids (HAL) and nitrohumic acids (NHA) from the Pakistani lignite coal. It was documented that under different extraction condition, the yield of HAL extracted with NaOH was 18%. While NHA regenerated with 5, 10, 15% HNO_3_ and extracted with KOH showed the yield of 25.5, 33 and 48% respectively^12^. Fatima et al.^43^ reported that bituminous and lignite sample treated with 0.5%, 1.5%, and 2.5% concentration of KOH produced 7.54%, 11.38%, and 17.98% yield of humic acid from bituminous coal whereas 9.98%, 13.45%, and 21.89% from lignite coal respectively. Zara et al.^23^ reported a maximal yield of 26.4% using a KOH concentration of 3.5%. Sabar et al.^44^ conducted an experiment on the effect of chemical pretreatments (nitric acid and hydrogen peroxide) with a fungal isolate from coal on the depolymerization of Pakistani subbituminous coal. The humic acid production was increased by the chemical pretreatment from 13.5 to 54.2% for nitric acid and 45.7% for hydrogen peroxide. Doskocil et al.^13^ conducted a study on comparison of humic acid isolated from European coal basins by the process recommended by International Humic Substances Society. It was reported that humic acids isolated from South Moravian and K1 lignites showed the highest yield of 11.4% and 11.1% whereas in contrast, the lowest yields (3.0% and 3.2%) were obtained from humic acids isolated from Maritza East and Kostolac lignites. Based on the above cited literature, it is worth mentioning that yield of humic acid depends on the source and experimental condition of isolation. The low content of humic acid appears to be less suitable as a source of humic acid for agriculture purpose. In view of that, there is some chemical ways that increases the production of humic acid. One of the best ways is to oxidize the coal by H_2_O_2_ that could disrupt the high molecular weight content of organic matter in coal and increase the amount of oxygen carrying functional groups. It would lead to increase in solubility of organic matter that increases HA production^45^.

### Proximate analysis

A summary of the results of the proximate analysis, including moisture content, volatile matter, ash content, and fixed carbon are summarized in Table 2. The moisture and volatile matter are gases and vapours driven off during pyrolysis, ash content is the inorganic residue left over after combustion and the fixed carbon is the non-volatile portion of coal^46^. This analysis is offered as a collection of test procedures that have been frequently employed as the basis for coal characterization in relation to coal utilisation. The MNMM has more moisture and ash content and less content of volatile matter and fixed carbon. In addition to that P-HAS has more moisture, ash content, fixed carbon and lower volatile matter than M-HAS.

### Elemental analysis

The elemental composition and their atomic ratios (Table 3) are comparable with reported values of various groups of coal derived HAs^47^. Atomic ratios have been used for the examination of structural changes of humic substances. This is because, it is an important indicator for degree of aromatic condensation and maturity of HAs and its derivatives^12^. The lignite coal and the HAs mainly comprises of carbon and oxygen as the lignite sample has 53–57% of carbon, 37–43% of oxygen and HAshas 56–58% of carbon and 35–39% of oxygen. Mukherjee et al. (2010) reported that the lignite sample collected from Nevyeli lignite mine of Tamilnadu, India had the carbon content of 69.40% and oxygen content of 24.4%. The carbon content of lignite sample of the present study was lower and oxygen content was higher than the content of lignite sample collected from nevyeli mines. Sabar et al.^44^, reported that humic acid extracted from Pakistan sub-bituminous coal had carbon content of 47.09–51.33%, hydrogen content of 3.07–4.22%, nitrogen content of 1.44–3.62%, oxygen content of 36.95–42.1% and S content of 4.12–6.04%. Hence, it is seen that in comparison to present study, the HAs has lower content of carbon and higher content of oxygen. Generally, coal that has higher component of carbon and lower component of oxygen contains lower amount of HA and it is utilized in fuel applications. The amount of nitrogen, hydrogen and oxygen decreases and its carbon content decreases as coal’s rank and age increases^37^. The extracted humic acid’s composition reported in the present study was found similarity with the results reported by Enev^48^ and Martins et al.^49^. The carbon, nitrogen and hydrogen content of the P-HAS is high and oxygen content is low in comparison to M-HAS (Table 3). This may be due to difference in chemical composition, molecular structure and depositional environment of both the lignite. HAs has complex nature and to that only the characterization of the elemental composition is not enough. So, the calculation of atomic ratios is important as it is used to investigate the structural changes of humic substances. It indicates mainly the of degree of aromatic condensation and maturity of humic acid and its derivatives^12^. The C/N ratio is considered as the indicator of the origin of HA and degree of condensation. Higher C/N ratios imply higher levels of stability condensation and humification of the organic matter in soil. The C/N ratio of the extracted HAs (P-HAS-74.67 and M-HAS-138.09) was higher than that of STD-HAS (29.84) which indicates that the extracted humic acid had higher stability, humification and condensation degree than STD-HAS. The H/C ratio is inversely related to condensation, aromaticity and humification degree of HAs^44^. Higher H/C values are a better sign of presence of large proportion of aliphatic compounds in the sample^34^. H/C ratio of P-HAS and M-HAS are different and it may be because of difference in aromatic moieties in the chemical structure of P-HAS and M-HAS. The H/C ratio of P-HAS (0.071) is more than that of M-HAS (0.033). It means that the P-HAS has less aromatic structure, lower degree of humification and a higher aliphatic structure of HA in contrast to that M-HAS i.e. it has more aromatic structure, greater degree of humification and a lower aliphatic structure of HA.O/C ratio represents oxygen containing content in organic materials i.e., carboxylic acids. The O/C ratio of P-HAS is slightly lower and M-HAS is slightly higher than the STD-HAS. It indicates that the P-HAS has higher amount of oxygen containing functional groups and M-HAS has lesser amount of oxygen containing functional groups than STD-HAS. In conclusion it can be stated that the higher O/C and H/C ratio might shows higher amount of –COOH and/or carbohydrates and lower degree of condensation and aromaticity respectively. According to the results of elemental analysis and atomic ratios, M-HAS is stable aromatic compound and has higher humification degree than STD-HAS and P-HAS that implies it can be used for commercial application.

**Table 3:** Elemental composition (% d.a.f), atomic ratios, mineral matter content, internal oxidation degree and aromaticity.

S. no	Entry	C	H	N	S	O^#^	C/N (d.a.f)	H/C (d.a.f)	O/C	%MM	Internal oxidation degree (ω)	Aromaticity (f_a_)
1	PM	56.92	4.72	0.80	1.42	36.14	71.15	0.082	0.634	15.14	1.229	0.599
2	MNMM	52.88	1.93	0.54	1.89	42.76	97.92	0.036	0.808	26.95	1.611	1.139
3	P-HAS	58.25	4.14	0.78	1.40	35.43	74.67	0.071	0.608	3.47	1.185	0.669
4	M-HAS	56.62	1.87	0.41	1.71	39.39	138.09	0.033	0.695	2.88	1.380	1.238
5	STD-HAS	56.41	3.40	1.89	0.00	38.30	29.84	0.060	0.678	-	1.398	0.201

*O*^*#*^ Calculated by difference.

*MM* Mineral matter (It is calculated by using Parr formula: MM = 1.08 × ash + 0.55 × sulphur), *ω* internal oxidation degree, *f*_*a*_ aromaticity.

The degree of aromaticity (f_a_) of the samples depends on H/C and C/H ratio (Table 3). H/C ratio indicates significant degree of aromatic character (i.e., the presence of benzene rings in the structure). It decreases with H/C ratio and increases with increase of C/H ratio. The calculated degree of aromaticity of M-HAS are more than of P-HAS. As the yield of both P-HAS and M-HAS were similar but degree of aromaticity is different and it may be because of difference in presence of benzene rings in the structure, aromatic and aliphatic character and degree of humification.

The internal oxidation degree calculation illustrates the origin and character of humic acid. The P-HAS, M-HAS and STD-HAS shown positive internal oxidation values that depicts the aerobic condition of the humic acid^38^. There is difference in the value of internal oxidation degree in P-HAS and M-HAS and it may be due to difference in origin and chemical characteristic of both the samples.

### Functional group determination

The content of total acidity, carboxylic and phenolic functional group in lignite and the extracted humic acid are listed in Table 4. In low rank coal, the phenolic and the carboxylic group content makeup a significant portion of total oxygen content. It is responsible for the ion-exchange characteristics of such coals and due so that these oxygen functional groups are of significant interest. These functional group and the associated cations greatly influence the properties of low rank coals and accordingly their behaviour in use^12^.

**Table 4:** Total acidity, carboxyl and phenolic groups (mmol/g) of the samples.

Samples	Acidity (mmol/g)		
	Total acidity	Carboxyl group (–COOH)	Phenolic group (–OH)
PM	5.20	3.90	1.30
MNMM	5.65	4.33	1.32
P-HAS	6.10	3.33	2.77
M-HAS	6.30	3.52	2.78

*–COOH* carboxylic groups, *–OH* hydroxyl group.

The carboxylate group determination on the basis of acid used for the removal of cations is satisfactory, provided on the basis of carboxyl groups present titrated with acid such as carbonate (CO3^2−^). Several researchers reported that the carbonate doesn’t occur in lignite and most of the brown coal because it has the incompatibility with acidic nature of such coals^12^. The M-HAS showed higher –COOH concentration than of P-HAS. It is significant to estimate these functional groups in the sample because the tested amount gives up accurate estimation of reactivity and humification rank of HAs. In the early stages of coalification, during humification there is an increase in phenolic groups and decrease in methoxyl, carboxyl and carbonyl groups contents^12^. Hatcher et al.^50^ uses a different analytical tool and shows that during the stages of coalification i.e., from lignin to lignite, chemical reaction enhances the cleavage of aryl ether bonds in lignin derivatives to produce phenols. It is observed that the carboxyl group content was higher than that of phenolic group in P-HAS and M-HAS which suggested that the HAs are originated from parent material of low-grade coal. The discovered total acidity of the extracted humic acid was agreed well with the outcomes reported by Nasir et al.^12^ for humic acid extracted from Pakistani lignite coal and Lakatos et al.^51^ for Hungarian brown coal derived humic acid.

### Spectroscopic techniques

#### SEM along with EDX

**Fig. 3:**
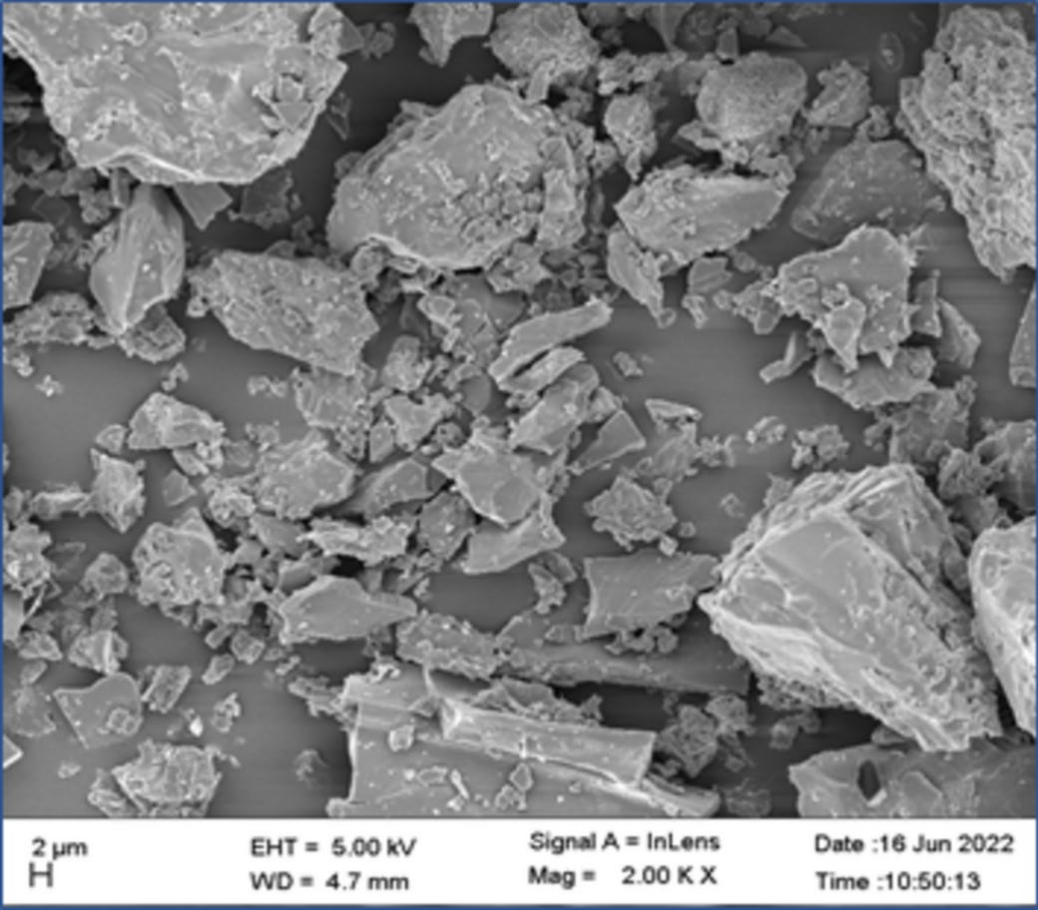
SEM images of a powdered lignite at 2 μm (2000 times magnification).

**Fig. 4:**
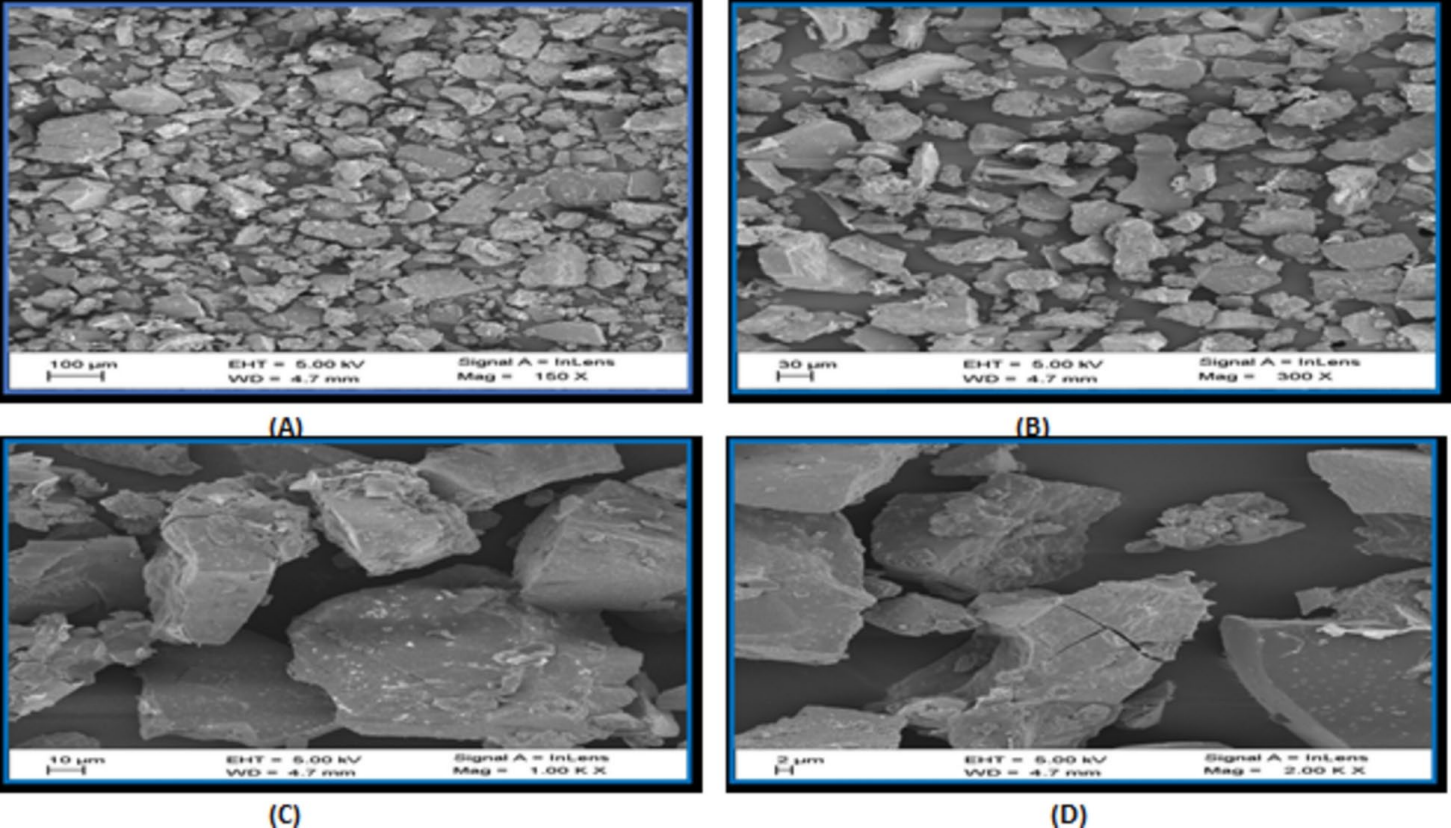
SEM images of a extracted humic acid from lignite of panandhro mines at (**A**) 100 μm (150 times magnification), (**B**) 30 μm (300 times magnification), (**C**) 10 μm (1000 times magnification), (**D**) 2 μm (2000 times magnification).

**Fig. 5:**
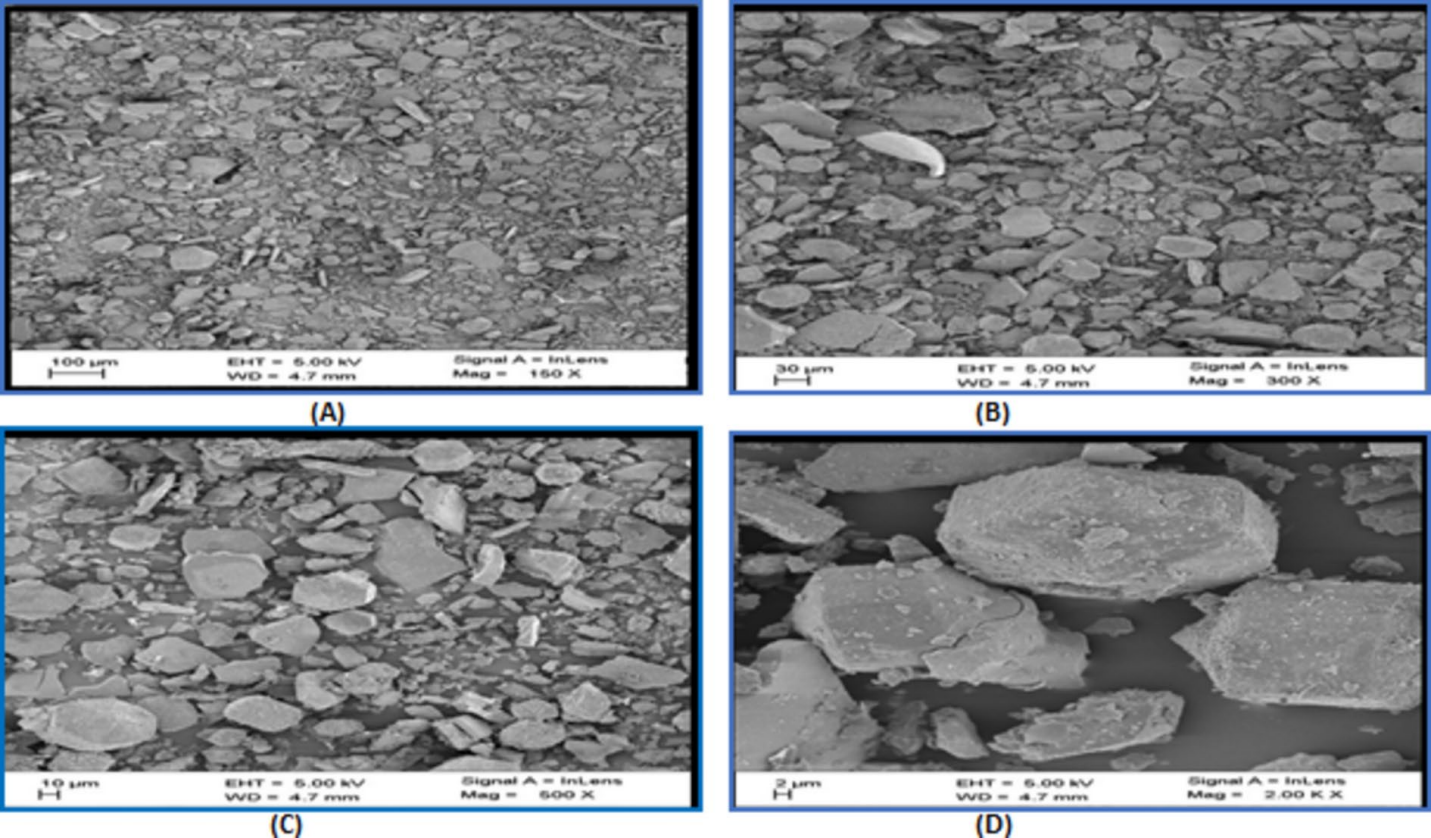
SEM images of a extracted humic acid from lignite of mata-no-madh mines at (**A**) 100 μm (150 times magnification), (**B**) 30 μm (300 times magnification), (**C**) 10 μm (1000 times magnification), (**D**) 2 μm (2000 times magnification).

Microstructural analysis of the powdered lignite and the HAs samples was performed using SEM^52^. The results are presented in Figs. 3, 4 and 5. The SEM study adequately indicated significant physical changes that shows that extracted HAs has some structural and morphological changes^43^. SEM images mainly hang on the pH and their synthesis and preparation conditions and include short rods, sheets and fibers that form a networks^41,53^.

HA particles, which typically range in size from (2–100 µm) are smaller and more uniform in size than lignite particles. At higher magnification (2000 times) (Fig. 4D), the particles of humic acid looks smooth, non-porous, globular like structure and has heterogeneous pores^43,54^. The forces that cause molecular aggregation can be weak such as the London and Van der Waals forces or strong such as charge transfer or hydrogen bonding^34^. The large sized molecule and the high structural compaction lead to strong bonds among the HAs molecules. Adhesion bonds between small molecules may contribute to the development of larger masses or the particles visible at low magnifications during the HA filtration process.

**Fig. 6:**
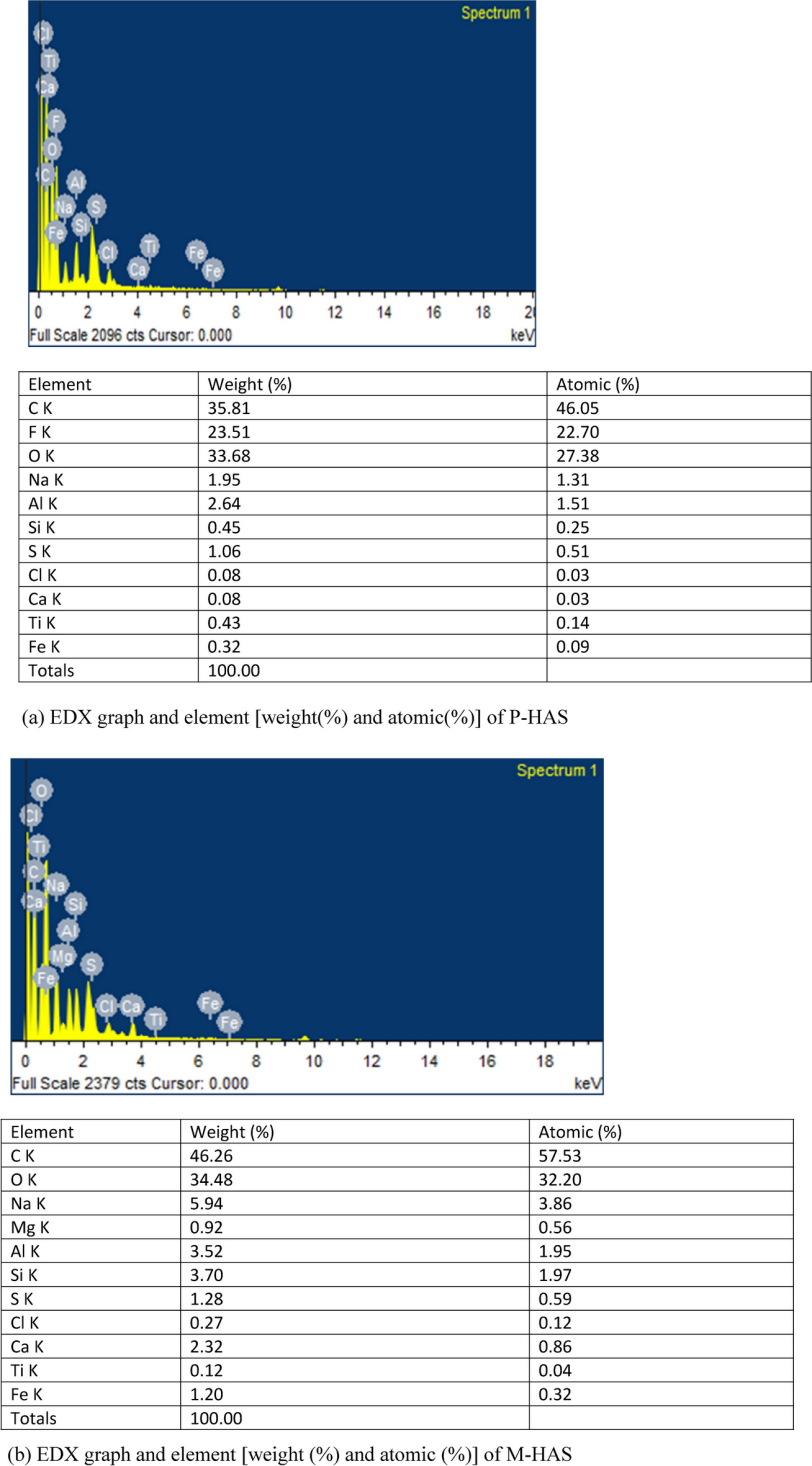
(**a**) EDX graph and element [weight(%) and atomic(%)] of P-HAS. (**b**) EDX graph and element [weight (%) and atomic (%)] of M-HAS.

EDX analysis determines the weight and atomic percentage (%) composition of the humic acid and it shows the presence of major elements like carbon (C), oxygen (O), fluorine (F), sodium (Na), aluminium (Al), silicon (Si), sulphur (S), chlorine (Cl), calcium (Ca), titanium (Ti) and iron (Fe) (Fig. 6a,b). P-HAS has more amount of carbon, oxygen and sodium whereas M-HAS has more amount of fluorine, aluminium, silicon, sulphur, chlorine, calcium, titanium and iron. It shows resemblance with average humic acid elemental composition. It has a generally wide range of molecular weights and is a comparatively high-molecular-weight substance in comparison to fulvic acid.

### UV–Vis spectroscopy analysis

**Fig. 7:**
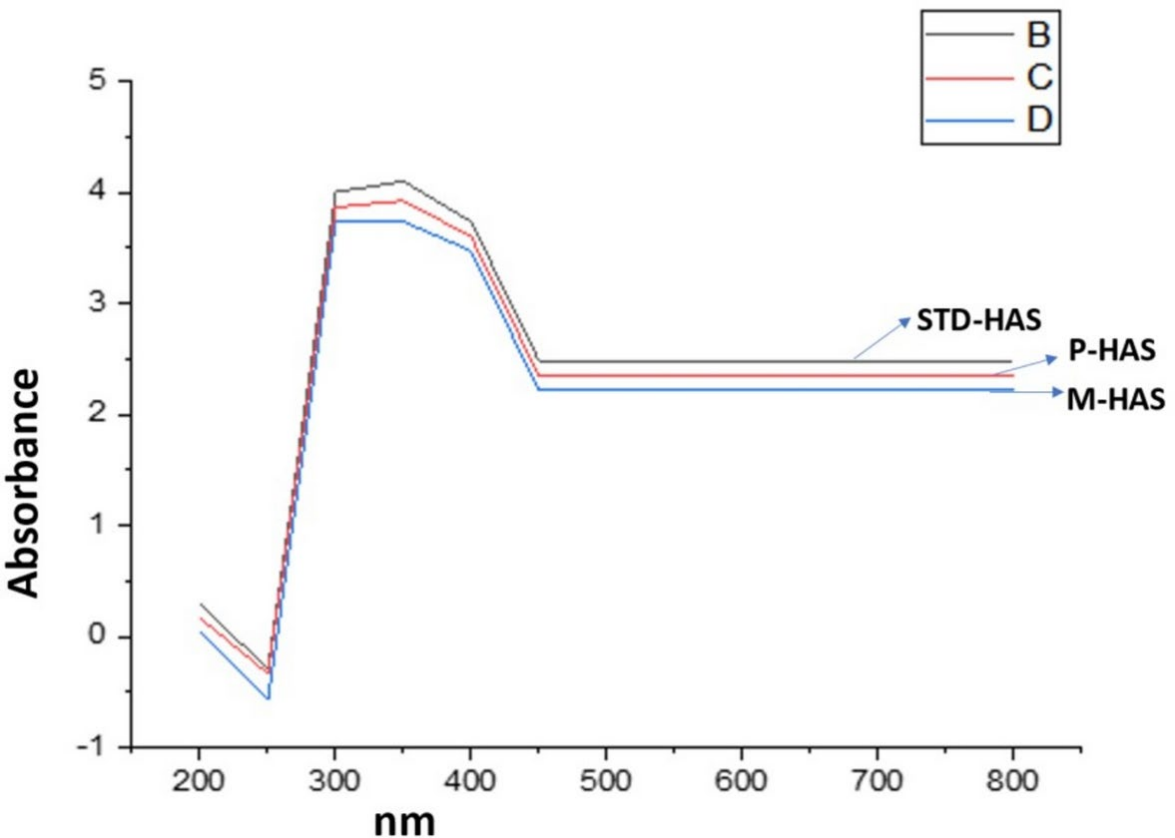
UV–Vis spectra of standard and extracted humic acid in which the legend B stands for STD-HAS, C stands for P-HAS, D stands for M-HAS.

In contrast to other molecular spectrophotometry techniques, UV–Vis spectroscopy falls under higher energy infrared radiation, takes into account molecular electronic energy transitions, while lower energy infrared radiation takes into account changes in molecular kinetic energy. The spectra of humic acid extracted from lignite (Fig. 7) exhibit peaks at about 220–300 nm which are caused by the presence of (π → π*) transition of electrons of aromatic groups and compounds of the lignin type^38,55–58^. Similar pattern of peaks were observed in the standard humic acid that is shown in Fig. 7.

The UV–Vis spectroscopic ratio at various wavelengths is an important humic acid characterising indication as it provides useful information on the composition and origin of organic matter. The main problem with this spectrometry is it is non-specific for analysing humic substances as it does not give band that is very characteristic of humic compounds^59^. In the UV–Vis range of spectrum, broad absorption bands are observed that implies different components (organic and inorganic components) might be present in the samples^35^. The spectra are influenced by chromophore group of humic substances and by the presence of solid particles. Within this context, some classical parameters such as humification index i.e. the ratio of E4/E_6_ (465 nm to 665 nm) and ∆log K (log Abs465-log Abs665) are most often used absorbance ratio that mirrors the molecular weight and is used for the identification of degree of humification, degree of condensation and degree of aromaticity^56,60^. Humic acids are characterized by the ratios below than 5. In general, when aromatic condensation and molecular mass increase, the ratio of E4/E6 decreases. Additionally, as the amount of oxygen increases, the ratio rises. Lower ratio indicates the presence of higher degree of condensation whereas the higher ratio reflects the low degree of condensation and of the aromatic humic compounds, it is reasonable to infer the existence of a significant amount of aliphatic structure^2,61^. The ratio has also relationship with the molecular weight of the humic acid. A higher ratio infers low molecular weight and a greater level of aliphaticity. However, a lower ratio indicates a higher molecular weight with greater level of condensation. It is mainly affected by pH, molecular size, concentration of free radicals, and content of O, C, CO_2_H and total acidity (HSA, Marc Pansu and Jacques Gautheyrou). The ratio also relates to the decomposition level and the presence of aromaticity. The E4/E6 ratio and log K was found to be highest in P-HAS than that of M-HAS and standard humic acid (Table 5). It indicates that the M-HAS has higher degree of condensation and aromaticity of the humic acid than that of P-HAS. The ∆ log K value is found to be < 0.6 (Table 5) that indicates the higher level of humification^56^.

**Table 5:** UV/Vis spectroscopic data.

S.no.	Samples	E4/E_6_	Δ Log K	Type of Humic acid
1	STD-HAS	1.01	0.01	Type-A
2	P-HAS	1.05	0.03	Type-A
3	M- HAS	1.02	0.01	Type-A

### Fourier transform infrared spectroscopy (FTIR)

**Fig. 8:**
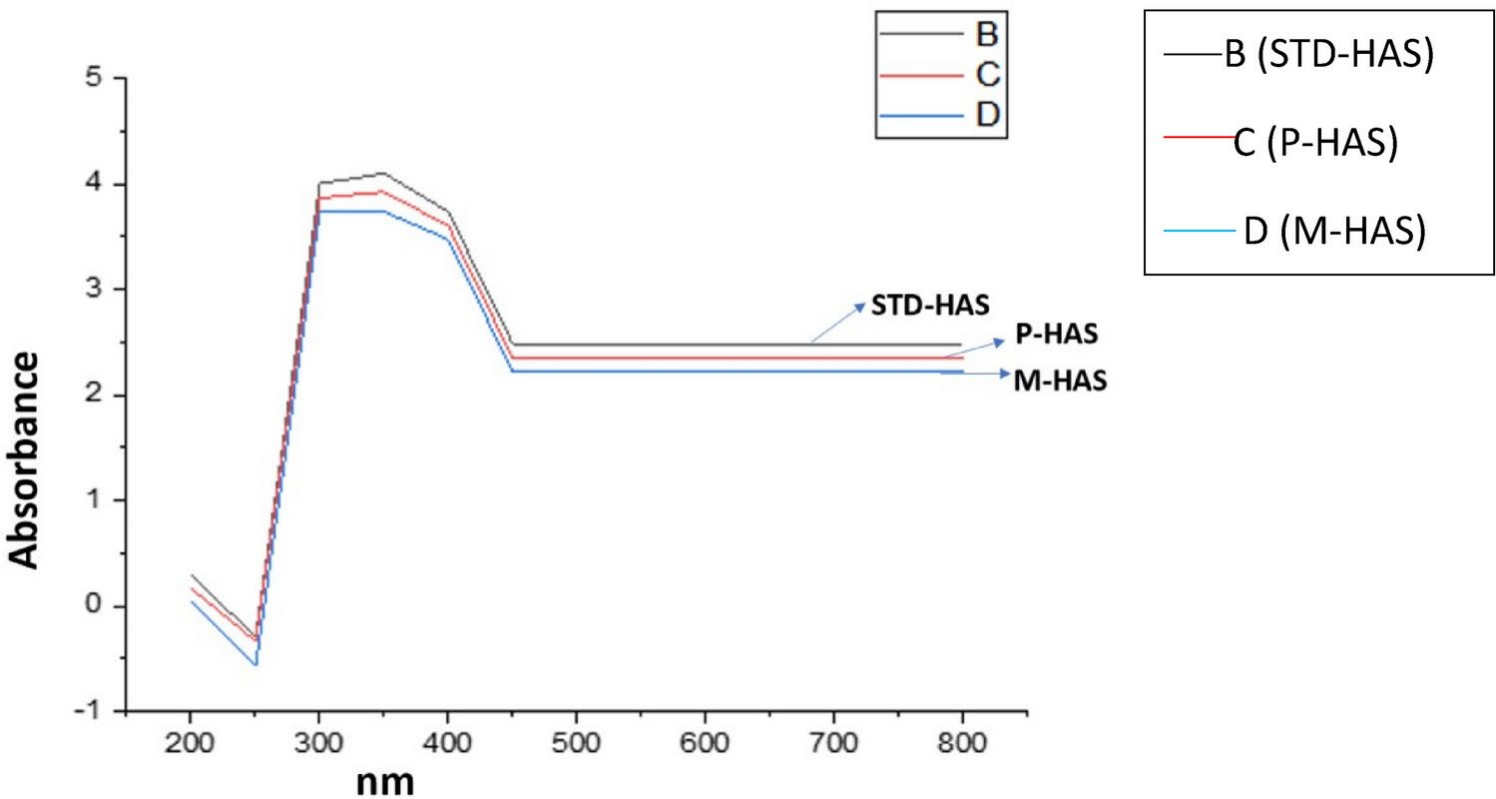
FTIR of isolated humic acid (HA) where legend D stands for STD-HAS, C stands for P-HAS, B stands for M-HAS.

Fourier transform infrared spectroscopy (FTIR) analysis is very useful tool for the determination of functional groups present in HAs. The validation of functional group by the use of qualitative FTIR analysis such as aliphatic structure, aromatic substuitions, hydrogen bond regions and 0-containing groups was conducted from 4000 to 500 cm^−1^ range values^62^. Generally, the lignite and HAs FTIR bands have common characteristics and distinctive vibrations, although the intensity of band vary depending on the lignite source and the utilised extraction solvent (de Souga and Braganca, 2018). In this study, FTIR spectra of STD-HAS, P-HAS and M-HAS are recorded and shown in Fig. 8. The assignment of major peaks is based on the published work of humic materials^53,63^. The spectra mainly represent the presence of oxygenated functional groups such as, –OH, –CO_2_H and C–O. The broad absorption band showed at around 3144 is the broadest peak and it is due to presence of O–H stretching (H bonded) which reflects the N–H stretching of amines and/or amides and the alcoholic/phenolic functional group^42^. High O/C and H/C ratios may be the cause of broader peak at the O–H stretching vibration^64^. There is absence of peak at around 3040 which is the representation of aromatic hydrogen band indicates that the structure is highly condensed and substuited. A peak at 2923 represents C–H stretching. The peak at 2900 represents aliphatic C–H stretching of CH_2_. A sharp band observed at around 1701.0 represents the stretching of C=O due to presence of carboxylic groups. The major second peak observed at 1620.04 is due to C=C, C=0, COO^−^ group and it attributes skeletal vibration of aromatic rings^57^. A couple of peaks at 1415 and 1330 represent C–O–H bending and O–H bending in plane which is a representation of carboxylic acid/ derivatives and alcohol/phenol groups. An aromatic structure with C–H out of plane deformation results in the assignment of a peak at 889. A small peak at 666 represents O–H bending out of plane and a peak at 412, 441 and 573 shows the vibration of Si–O^65^. These bands show the impurities of silicate^42^. The FTIR peaks demonstrate the extracted HAs have aromatic structure and contain aliphatic side chains. The successful extraction of HAs from samples of lignite may be confirmed by comparing the FTIR spectra of previously reported results^55–58^. The list of major infrared absorption bands for extracted humic acid (P-HAS and M-HAS) are shown in Table 6.

**Table 6:** Major infrared absorption bands (cm^-1^) for lignite extracted humic acid (P-HAS and M-HAS).

Sample	Absorption bands (cm^−1^)	Assignment	Functional class
Lignite extracted humic acid (P-HAS and M-HAS)	3144	O–H stretching (H bonded)/N–H stretching of amines	Alcholic/phenolic functional groups
	2900	COOH bonded hydrogen stretching	Aliphatic –CH component
	1701.0	Stretching of C=O	Presence of carboxylic group
	1620.04	C=C, C=O, COO– NH2 scissoring (1-amines)	Amines/carboxylic acid
	2923	Aliphatic stretching	Variation in aliphatic –CH component
	1415	C–O–H bending	Carboxylic acid/derivatives
	1330	O–H bending in plane	Alcohols/phenols
	889	Aromatic C–H bending	Aromatic structure
	666	O–H bending out of plane	Alcohol/phenols
	412,441 and 573	Si–O bond	Si–O group

## Conclusion

The result of the current investigation concludes that the humic acid can be extracted from lignite of two different mines of western region of Kutch district of Gujarat, India. The result showed that the yield of humic acid from Panandhro mines is 28.5% than that of Mata-no-madh mines is 26.4%. It is almost similar in both the samples which is due to similar extraction and refinement procedure. The extracted humic acid from both samples (P-HAS and M-HAS) has high level of humification and lies in the category of type-A humic acid. The H/C ratio of P-HAS is more than that of M-HAS which indicates M-HAS has higher saturation and aromaticity of organic compound. The M-HAS has higher aromaticity and condensation (lower E4/E_6_ ratio) and internal oxidation degree than P-HAS. This difference is may be due in difference in molecular weight, oxygen content, aromatic moieties of both the samples. The extracted HAs has positive values of internal oxidation degree which depicts the aerobic condition of the HAs. The total acidity of M-HAS is more in comparison to P-HAS and carboxylic acidity was more favourably formed than that of hydroxyl acidity. FTIR, UV/Vis and SEM/EDX proved to be useful technique for the study of chemical structure of lignite and its derivatives. The results showed that the extracted HAs has similarity in characteristics with standard HAs.

Therefore, the results of this study will be helpful in the optimization of western Indian lignite and its derivatives. The data will also make contribute in the understanding of chemical structure of lignite and its alkaline extract for specific purposes. In conclusion, it can be stated that the findings of this work can provide basic and relevant knowledge required for the future environment- friendly extraction from the potential raw materials and its waste. It provides a simple reference to develop future application of HAs as green fertilizer for agricultural uses.

## Data Availability

The data that support the findings of this study are available from the corresponding author upon request and all the reported data are generated in labs of IIT(ISM) Dhanbad.
